# Which arthralgia patients benefit most in reduction of subclinical joint inflammation by methotrexate treatment: results from the TREAT EARLIER trial

**DOI:** 10.1136/rmdopen-2025-006102

**Published:** 2026-01-20

**Authors:** Stijn Claassen, Quirine A Dumoulin, Herman K Glas, Esmeralda Molenaar, Hanna W van Steenbergen, Annette HM van der Helm - van Mil

**Affiliations:** 1 Rheumatology, Leiden University Medical Center, Leiden, The Netherlands; 2 Rheumatology, Reumazorg Zuid West Nederland, Goes, The Netherlands; 3 Rheumatology, Groene Hart Hospital, Gouda, The Netherlands; 4 Rheumatology, Erasmus Medical Center, Rotterdam, The Netherlands

**Keywords:** Arthritis, Rheumatoid, Risk Factors, Methotrexate, Therapeutics

## Abstract

**Objectives:**

The TREAT EARLIER trial showed in clinically suspect arthralgia (CSA) that methotrexate induced reductions in subclinical inflammation and related disease burden during the year of treatment, which was sustained thereafter. We studied whether the treatment response defined at the level of subclinical joint inflammation was present in all treated CSA patients and, if not, what characterises the subgroup of responders.

**Methods:**

CSA patients with subclinical inflammation were randomised to receive an intramuscular glucocorticoid injection and a 1-year course of methotrexate. Treatment response was defined as a reduction of MRI-detected synovitis, tenosynovitis or osteitis levels beyond the smallest detectable change at 12 months. Baseline clinical and imaging characteristics were studied in relation to treatment response. Predictive values were determined.

**Results:**

44 of 115 (38%) treated patients had an MRI-defined treatment response. These patients also significantly improved in pain and physical functioning (−22 Visual Analogue Scale pain, −0.29 Health Assessment Questionnaire). Baseline clinical variables were not independently associated with this response, in contrast to the severity of subclinical joint inflammation. Tenosynovitis and osteitis levels in particular were predictive. Patients with ≥2 sites with tenosynovitis or a combination of osteitis and tenosynovitis (with ≥1 of these features at ≥2 sites) had high positive predictive values (PPV 77%, 79%). PPVs were similar in ACPA-positive and ACPA-negative patients at increased risk for rheumatoid arthritis (RA).

**Conclusions:**

CSA patients with subclinical inflammation who responded best to methotrexate during the first year were identified by increased levels of subclinical inflammation at diagnosis, primarily due to multiple sites of tenosynovitis with/without osteitis. These data may contribute to personalised medicine for arthralgia at risk for RA.

**WHAT IS ALREADY KNOWN ON THIS TOPIC**

- The TREAT EARLIER trial showed in clinically suspect arthralgia (CSA) that methotrexate induced reductions in subclinical inflammation and related disease burden during the year of treatment, which was sustained thereafter.
- This finding was made at the group level. However, it is important to know whether all treated patients experienced improvements, or whether, as in the treatment of classified rheumatoid arthritis (RA), a subgroup of patients experienced significantly greater benefits than others. In this case, it would be important to identify these responding patients.

**WHAT THIS STUDY ADDS**

- Treatment response, as measured by a reduction in severity of subclinical joint inflammation during the first year, occurred in a subgroup of patients (38%); these patients also showed a significantly better response in pain, morning stiffness and disability.
- These patients can be recognised by the severity of subclinical inflammation at baseline, especially by having tenosynovitis of 2 or more tendon sheaths or a combination of osteitis and tenosynovitis (of which one has at least two affected tendon sheaths or bones).

**HOW THIS STUDY MIGHT AFFECT RESEARCH, PRACTICE OR POLICY**

- When the aim is to treat CSA to reduce the disease burden, treating only the subgroup of patients with tenosynovitis of 2 or more tendon sheaths or a combination of tenosynovitis and osteitis can prevent overtreatment and promote personalised medicine for individuals with arthralgia at risk for RA.

## Introduction

 Rheumatoid arthritis (RA) patients have a significant disease burden that is related to chronic inflammation and expressed by symptoms, disability and loss of work productivity.^1^,^4^ These symptoms and limitations already develop before clinically inflammatory arthritis manifests.^1 5 6^ It is assumed that autoimmune processes and subclinical inflammation in the symptomatic prearthritis risk stage are still reversible.^7^ Indeed, several recent proof-of-concept trials in arthralgia at risk for RA and subclinical joint inflammation showed promising results of treatment with methotrexate and abatacept.^8^,^10^

One of these studies is the TREAT EARLIER trial, which evaluated the efficacy of a 1-year course of methotrexate in patients with clinically suspect arthralgia (CSA) and subclinical joint inflammation.^8^ Treatment resulted in sustained improvements in subclinical joint inflammation, symptoms and physical impairments that were induced during the year of treatment and were sustained in the second year without treatment. Thus, potential treatment goals in patients who are at risk of RA are not only aimed at reducing the progression to RA, but also at reducing the inflammatory burden of at-risk individuals. Treatment was also shown to be cost-effective, largely due to improved work productivity in the treated group.^11^ These benefits were observed across the total trial population over a 2-year follow-up, regardless of progression to RA, suggesting that early intervention is useful to reduce the inflammatory burden in all at-risk individuals with arthralgia. Additional analyses of the 2-year trial data showed that the improvement in inflammatory burden was present in ACPA-positive and ACPA-negative patients at increased risk for RA, but not in ACPA-negative patients who retrospectively had a low risk for RA.^12^ These results show that it is important to reduce heterogeneity in risk by excluding patients with a low disease risk, as patients with a low risk for a disease are not expected to have any treatment benefit. Furthermore, the main results and additional results during 2 years of follow-up on inflammatory disease burden suggest that treating all individuals with CSA who are at increased risk for RA is beneficial in reducing inflammatory burden.^8 12^

However, it is still unknown whether indeed all treated patients responded or whether CSA patients vary in their degree of treatment response regarding inflammatory burden reduction. It is possible that, as in the treatment of classified RA, a subset of patients experienced significantly greater benefits than others. For the at-risk phase, this would imply only those patients should receive treatment when disease burden reduction is the aim. To date, no studies have been conducted into treatment response on the level of subclinical inflammation in the at-risk stage of CSA to understand who responded best to treatment. This knowledge is essential for identifying the population most likely to benefit from treatment, helping to prevent overtreatment without denying treatment to those who need it most.

Therefore, we aimed to investigate whether all patients in the TREAT EARLIER trial responded to treatment by a reduction of disease burden to methotrexate. We defined treatment response during the year of treatment on the level of subclinical inflammation since this is an objective measure, but we also studied the relation between an imaging-detected response with improvements in patient-reported inflammatory burden expressed as joint pain, morning stiffness and functional disability. Second, we aimed to identify the characteristics of those who showed the greatest treatment response. We first investigated the total TREAT EARLIER population and, in sub-analyses, we evaluated the ACPA-positive and ACPA-negative increased-risk patients separately.

## Methods

### Patients

The TREAT EARLIER trial is a randomised, double-blind, placebo-controlled proof-of-concept trial that recruited 236 patients (119 treatment arm, 117 placebo arm) in all rheumatology outpatient clinics in the southwest region of the Netherlands. The protocol and amendments were approved by the LUMC medical ethics committee and previously described elsewhere.^8^ Written informed consent was obtained from all participants in accordance with the Declaration of Helsinki. Patient partners were involved in the design of the TREAT EARLIER trial protocol.

Adults aged ≥18 years with arthralgia at risk of developing RA were eligible. We used a two-level definition to identify patients predisposed to developing RA. First, patients needed to have recent-onset (within the past year) arthralgia suspected of progressing to RA according to the treating rheumatologist (ie, CSA). Second, a unilateral contrast-enhanced MRI scan of their most painful side—or dominant side if symptoms were equally severe—had to show subclinical joint inflammation in the hand or forefoot. Subclinical joint inflammation was considered to be present if at least one joint showed synovitis, tenosynovitis or osteitis according to two independent readers and was present in ≤5% of age-matched, symptom-free volunteers of the same age at the same location. Readers of MRI scans were masked to clinical data and showed strong intrareader reliability (intraclass correlation coefficient 0.92–0.99) and inter-reader reliability (0.91–0.98). Further details on participants, randomisation and masking procedures are provided in online supplemental files 1 and 2.

In this analysis, we studied the patients from the treatment arm who had a repeated MRI in the first year (see below) to find what characterises patients who respond best to methotrexate.

### Treatment

Active treatment consisted of a single intramuscular glucocorticoid injection (120 mg methylprednisolone) on inclusion, followed by a 1-year course of methotrexate (titrated to 25 mg/week or the highest tolerated dose over 4 weeks). Placebo consisted of a single placebo injection followed by a 1-year course of placebo tablets. All patients received folic acid supplementation (5 mg/week).

### Follow-up

Trial visits occurred every 4 months during the first 2 years of follow-up. An immediate additional visit was scheduled in case of increasing symptoms between two trial visits. Clinical and questionnaire data were collected at every visit, including physical functioning (via the Health Assessment Questionnaire Disability index (HAQ-DI), scored at 0–3), joint pain and general health on the Visual Analogue Scale (VAS, scale 0–100) and morning stiffness duration (in minutes). Contrast-enhanced MRIs of metacarpophalangeal (MCP) joints, metatarsophalangeal (MTP) joints and wrist joints were done at baseline, 4 months, 12 months and 24 months.

### MRI

MRI was captured by a 1.5 T extremity scanner with contrast enhancement, scanning the (MCP 2–5), wrist and (MTP 1–5) joints. Two readers independently assessed the images on subclinical inflammation (synovitis, tenosynovitis and osteitis) using the RA MRI scoring (RAMRIS) method.^13^ Osteitis was scored on a scale 0–3 based on the affected volume of the bone (no osteitis, >0%–33%, >33%–66%, >66%) and synovitis was scored on a range 0–3 based on the volume of enhancing tissue in the synovial compartment (none, mild, moderate, severe).^13^ The tenosynovitis score was based on the thickness of peritendinous effusion or tenosynovial proliferation with contrast enhancement in a range of 0–3 (none, <2 mm, 2–5 mm, >5 mm).^14^ Readers of the MRIs during the follow-up were blinded to any clinical data and had excellent intrareader and inter-reader reliability (intraclass correlation coefficients >0.90). Serial MRIs were scored with known time order, blind to clinical characteristics, study endpoints or treatment allocation. Further details on the MRI measurements and scoring methods are provided in online supplemental file 3. Of the 119 participants in the treatment arm, 115 had a repeated MRI in the first year.

### Definition of treatment response

Treatment response was defined as a reduction of MRI-detected subclinical inflammation of the hand and forefoot between baseline and 12 months (end of treatment period). In order to discern measurement variability and noise from a ‘true’ reduction of subclinical inflammation, we applied the smallest detectable change (SDC) as a cut-off for the definition of response (online supplemental file 4, figure S4). In line with the literature, the SDC was estimated using the SD of the differences between change scores of two readers in the complete TREAT EARLIER trial, using the following formula: $SDC=1.96\times \frac{{SD}_{∆\left(CHANGE-SCORES\right)}}{\sqrt{2}\times \sqrt{k}}$ where k represents the number of readers averaged (here, k=2).^15^

The primary definition of treatment response was achieving a reduction of inflammation on the level of either synovitis, tenosynovitis or osteitis beyond the SDC at 12 months. The calculated SDCs for the individual components of subclinical inflammation were 1.6 for synovitis, 1.7 for tenosynovitis and 1.5 for osteitis. In a sensitivity analysis, treatment response was defined as a reduction of subclinical inflammation beyond the SDC of the total RAMRIS-inflammation score (sum of tenosynovitis, synovitis and osteitis). The corresponding SDC for total inflammation was 3.2 RAMRIS-units. To evaluate the robustness of these associations, the same analyses were repeated in the placebo arm, under the hypothesis that the predictive variables would not show the same associations in the absence of treatment.

We evaluated whether this imaging-based definition of treatment response was associated with improvements in disease burden. Therefore, we compared the improvements in functional disability (HAQ-DI scores), joint pain (VAS 0–100) and morning stiffness duration (minutes) in 12 months between treatment responders and non-responders. If data at 12 months was unavailable due to loss to follow-up or reaching the endpoint of clinical arthritis prior to the 12 month mark, data from the previous visit was used (last observations were carried forward).

### Statistical analysis

The analyses were performed according to the intention-to-treat principle, assuming that all participants had taken the study medications according to the protocol. Patients with/without MRI-defined treatment response were compared for the change in severity of pain, morning stiffness duration and disability using Student’s t-tests.

To identify predictors, clinical and imaging characteristics at baseline that were expected to hold information on treatment response based on previous research were compared using univariable logistic regression, with correction for multiple comparisons using the Benjamini-Hochberg method. The studied clinical predictors were: morning stiffness >60 min, difficulty making a fist, positive squeeze test, grip strength, tender joint count, ACPA-positivity, RF-positivity, increased C reactive protein (CRP) and total HAQ-DI score, since they are associated with subclinical inflammation and/or RA development in CSA patients in previous studies.^16 16^,^19^ The imaging variables were total inflammation score and the components (synovitis/tenosynovitis/osteitis). Variables with a significant association (p<0.05) were included in the multivariable logistic regression model.

Positive and negative predictive values (PPV and NPV) of identified predictors were calculated. In subanalyses, this was also done for ACPA-positive and ACPA-negative patients at increased risk for RA separately. ACPA stratification was performed because the pathophysiology of ACPA-positive and ACPA-negative disease development is different, making it possible that treatment effects may also differ.^20^ Furthermore, risk stratification was not possible at trial design in 2014 but has recently advanced. This allowed us to identify the patients who were retrospectively at low risk for RA. These were excluded from the subanalyses, because patients at low risk theoretically cannot benefit from preventive treatment, which was indeed shown in recent analyses of the 2-year data.^12^ Thus, the subanalyses reduced the heterogeneity in underlying pathophysiology and disease risk before studying the treatment response.

## Results

### Baseline characteristics

Table 1 presents the baseline characteristics of the 115 studied patients. The mean age was 46.5 years (SD 13.1), and 71% were female. The median symptom duration was 17 weeks (IQR 7–35), and 26% of patients were ACPA-positive.

**Table 1:** Baseline characteristics

	All patients (115)	MRI-defined treatment response – (71)	MRI-defined treatment response + (44)
Clinical characteristics			
Age in years, mean (SD)	46.5 (13.1)	45.7 (12.4)	47.8 (14.3)
Female, n (%)	71 (62)	45 (63)	26 (59)
Symptom duration in weeks, med (IQR)	17 (7–35)	21 (9–39)	13 (6–26)
Morning stiffness >60 min, n (%)	39 (36)	19 (28)	20 (49)
Patient reported joint swelling, n (%)*	61 (54)	34 (49)	27 (61)
Positive squeeze test of MCP joints, n (%)	50 (43)	33 (47)	17 (39)
Decreased grip strength, n (%)*	99 (88)	57 (82)	42 (96)
Difficulties making a fist, n (%)*	18 (16)	11 (16)	7 (16)
Tender joint count (68 joints), median (IQR)	4 (1–8)	4 (1–8)	4 (1–8)
Smoking currently, n (%)	19 (71)	11 (16)	8 (20)
Increased CRP, n (%)	35 (31)	22 (32)	13 (30)
Rheumatoid factor positive (>3.5 IU/mL), n (%)	32 (28)	20 (28)	12 (27)
ACPA positive (>7 IU/mL), n (%)	30 (26)	19 (27)	11 (25)
HAQ-DI total score, med (IQR)	0.63 (0.13–1.1)	0.50 (0.13–1.0)	0.88 (0.38–1.4)
MRI characteristics			
Total inflammation score, med (IQR)	6 (3.5–9)	3.5 (2.5–6.5)	9 (6.6–13.9)
Tenosynovitis score, med (IQR)	1.5 (0–3.5)	1 (0–1.5)	3.8 (1.6–7.8)
Synovitis score, med (IQR)	2.5 (1.5–4)	2 (1–3)	3 (2–5.5)
Osteitis score, med (IQR)	1 (0–2.5)	1 (0–2)	1.3 (0.5–3.0)
Tenosynovitis sites, med (IQR)	1 (0–2)	0 (0–1)	2 (1–4)
Synovitis sites, med (IQR)	1 (0–1)	0 (0–1)	1 (0–2)
Osteitis sites, med (IQR)	0 (0–1)	0 (0–1)	0 (0–1)

* In either hand.

CRP, C reactive protein; HAQ-DI, Health Assessment Questionnaire Disability Index; MCP, metacarpophalangeal.

### Treatment response

44 out of 115 (38%) patients showed an MRI-defined treatment response after 1-year course of methotrexate. Exploration of whether patients experienced improvement in synovitis, tenosynovitis and/or osteitis revealed that many patients showed improvement in ≥1 inflamed tissue, with the most frequent improvement in tenosynovitis (online supplemental file 5, figure S5).

### Treatment response on MRI was accompanied by improvement in patient-reported outcomes

**Figure 1:**
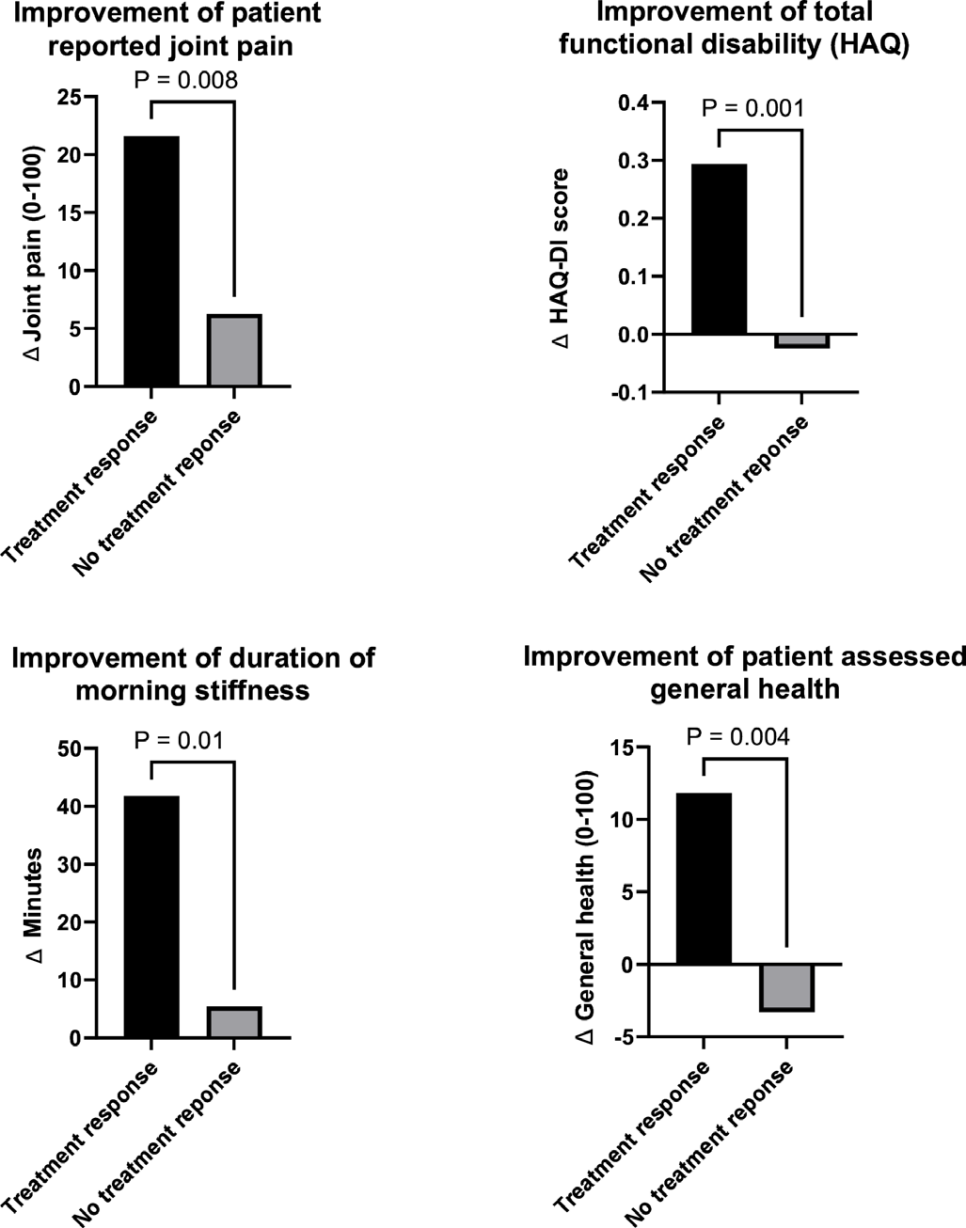
Improvement of joint pain and functional disability compared with baseline after a 1-year course of methotrexate treatment, comparing patients with and without MRI-defined treatment response. HAQ-DI, Health Assessment Questionnaire Disability Index.

To evaluate whether the definition of treatment response was associated with improvements in disease burden in the same year, we compared the improvements in disability, joint pain and morning stiffness between MRI-defined treatment responders and non-responders. Patients with treatment response showed, compared with non-responders, significant improvements; they had reduced morning stiffness (mean improvement in duration 42 min vs 5 min, p=0.01), better physical function (mean improvement of 0.29 HAQ-DI total score vs +0.02, p<0.01), reduced patient-reported joint pain (mean improvement of 22 vs 6 on a 0–100 scale, p<0.01) and improved patient-assessed general health (mean improvement of 12 vs a +3 on a 0–100 scale, p<0.01) (figure 1). Thus, treatment response at imaging level corresponded to improvements in symptoms and physical impairments.

### Baseline clinical features in relation to treatment response

Of the studied clinical variables (table 2), only increased functional disability as measured by the HAQ-DI score (OR 2.4, 95% CI 1.2 to 4.5) and morning stiffness of joints >60 min (OR 2.4, 95% CI 1.1 to 5.4) were univariably associated with treatment response. After correction for multiple testing, only HAQ remained significantly associated. In a multivariable model including HAQ and morning stiffness, none remained significantly associated.

**Table 2:** Univariable and multivariable logistic regression analysis of baseline characteristics as predictors of MRI-defined treatment response

		Univariable OR (95% CI)	Multivariable OR (95% CI)
Clinical characteristics			
Morning stiffness >60 min		2.4 (1.1 to 5.4)*	2.1 (0.88 to 4.8)
Positive squeeze test of MCP joints		0.7 (0.33 to 1.6)	
Decreased grip strength		4.4 (0.94 to 20.8)	
Difficulties making a fist		1.0 (0.37 to 2.9)	
Tender joint count (68 joints)		1.0 (0.93 to 1.1)	
Increased CRP		0.90 (0.39 to 2.0)	
ACPA positive (>7 IU/mL)		0.91 (0.39 to 2.2)	
Rheumatoid factor positive (>3.5 IU/mL)		0.96 (0.41 to 2.2)	
HAQ-DI total score		2.4 (1.23 to 4.5)†	2.0 (0.97 to 3.9)
MRI characteristics			
Total inflammation score		1.4 (1.2 to 1.7)†	
Tenosynovitis score			2.0 (1.4 to 2.8)*
Synovitis score			0.96 (0.68 to 1.4)
Osteitis score			1.5 (1.2 to 2.0)*
Number of sites of tenosynovitis and osteitis			
Tenosynovitis	0		Reference
(per sheath)	1		2.1 (0.6 to 8)
	2		12 (3 to 45)*
	>3		70 (12 to 397)*
Osteitis	0		Reference
(per bone)	1		1.6 (0.5 to 5)
	>2		8 (1.7 to 40)*

* Significant at p<0.05.

† Significant after correcting for multiple comparisons using Benjamini-Hochberg.

CRP, C reactive protein; HAQ-DI, Health Assessment Questionnaire Disability Index; MCP, metacarpophalangeal.

### Baseline MRI features in relation to treatment response

When examining the imaging variables, the baseline total inflammation score was associated with treatment response, also after correction for multiple testing (p<0.001). For each increase of one RAMRIS unit, the odds of treatment response rose by 43% (OR 1.4, 95% CI 1.2 to 1.7).

To further characterise which inflamed tissue contributed to this finding, the severity of inflammation in the three different inflamed tissues (tenosynovitis, synovitis and osteitis) was studied in a multivariable analysis. Both tenosynovitis and osteitis scores were independently associated with treatment response (table 2) (OR 2.0, 95% CI 1.4 to 2.8 and OR 1.5, 95% CI 1.2 to 2.0), whereas synovitis was not. Thus, the severity of tenosynovitis and osteitis at treatment start is positively associated with a treatment response.

The severity of tenosynovitis and osteitis was measured with RAMRIS. To translate these results into clinically interpretable variables, we studied the number of inflamed tendon sheaths and bones. A dose-response relationship was observed, with a higher number of inflamed sheaths or bones associated with a greater likelihood of response to treatment. More specifically, patients with ≥2 inflamed tendon sheaths or ≥2 bones with osteitis had significantly increased odds of a decrease in subclinical inflammation after treatment (table 2).

We also explored whether a combination of tenosynovitis and osteitis locations was associated with treatment response (table 3). Patients with ≥2 tendon sheaths and ≥1 bone(s) with inflammation, or ≥2 bones and ≥1 tendon sheath(s), had significantly increased odds of treatment response. Patients with ≥1 tendon sheath(s) and ≥1 bone(s) with inflammation also had a statistically significant increased OR, although lower.

**Table 3:** Combinations of tenosynovitis and osteitis sites and their association with MRI-defined treatment response

Combinations of MRI characteristics	OR (95% CI)
Osteitis >1 and tenosynovitis >1	4 (1 to 11)*
Osteitis >2 and tenosynovitis >1	9 (1 to 80)*
Osteitis >1 and tenosynovitis >2	10 (2 to 49)*
Osteitis >2 and tenosynovitis >2	Not calculable†

ORs signify the odds of having a treatment response for patients with a certain combination of inflamed joint tissues in comparison to patients who do not have that combination.

* Statistically significant with p<0.05.

† OR is not calculable since all patients with this combination showed MRI-defined treatment response.

Thus, having two lesions of either tenosynovitis or osteitis, or having a combination of both, was predictive for treatment response at baseline.

### Predictive values

Subsequently, the PPV and NPVs of these characteristics were determined (table 4). The PPV of having ≥2 tendon sheaths with tenosynovitis was 77%, indicating that 77% of CSA patients with two or more locations of tenosynovitis showed a treatment effect after a 1-year course of methotrexate (table 4). Having two or more locations of osteitis and at least one location of tenosynovitis or vice versa at baseline showed a PPV of 79%.

**Table 4:** Positive and negative predictive values of number of tenosynovitis and osteitis locations, below stratified for ACPA status

Characteristics	Positive predictive value	Negative predictive value
Tenosynovitis of one or more locations	56%	84%
Tenosynovitis of two or more locations	77%	82%
Tenosynovitis of three or more locations	91%	74%
Osteitis of two or more locations	67%	65%
Combination of osteitis and tenosynovitis*	79%	67%

Stratified for ACPA status	ACPA Positive	ACPA negative increased risk	ACPA positive	ACPA negative increased risk
Tenosynovitis of one or more locations	48%	73%	100%	56%
Tenosynovitis of two or more locations	73%	89%	100%	63%
Tenosynovitis of three or more locations	78%	100%	78%	55%
Osteitis of two or more locations	100%	60%	68%	32%
Combination of osteitis and tenosynovitis*	100%	63%	79%	33%

* Combination of osteitis and tenosynovitis, where at least one involves two or more sites.

### Subanalyses predictive values in ACPA-positive and negative patients at increased risk for RA

In subanalyses, the predictive values were also studied separately in ACPA-positive and ACPA-negative patients at increased risk for RA. As previously published, all ACPA-positive patients were at increased risk, and specifically 35 of 85 ACPA-negative patients (41%) included in this analysis were at increased risk.^12^ Excluding low-risk patients reduced the heterogeneity in the trial population before evaluating the predictive value of the predictors of treatment response. Within the ACPA-positive group, 37% showed an MRI-defined treatment response. Studying the identified predictors showed that ACPA-positive patients with two or more locations of tenosynovitis and osteitis had a high PPV for treatment response (table 3). The NPV was also high. Within the ACPA-negative patients at increased risk of RA, 66% had a treatment response. In this subgroup, the identified predictors also had a high PPV for predicting treatment response; however, their absence was less efficient in ruling out an MRI-defined treatment response (table 3). Together, these data show that also in these subgroups, part of the patients experienced an MRI-defined treatment response and that the patients with a response can be well recognised by having two or more locations of tenosynovitis and osteitis at treatment start.

### Sensitivity analysis

In a sensitivity analysis, the cut-off for treatment response was defined differently, now as reduction of the total inflammation score beyond the SDC of the total inflammation score. In this analysis, 34 out of 115 (30%) of patients showed a treatment response. The logistic regression analysis of baseline characteristics yielded very similar results, showing that the increased severity of tenosynovitis and osteitis is associated with treatment response in a dose-response relationship (online supplemental 6, table S6.1–S6.3).

Finally, analyses were done in the placebo arm. If an association between higher subclinical inflammation scores for tenosynovitis at baseline and a response in inflammation scores over time in the treatment group is purely based on higher baseline values, or if this observation is merely due to regression to the mean, we would expect to find the same relations in the placebo arm. When repeating the multivariable logistic regression analysis in the placebo arm, baseline tenosynovitis (the strongest predictor of treatment effect in our analysis) was not significantly associated with a reduction of subclinical inflammation beyond the SDC in the absence of treatment (OR 1.09, 95% CI 0.9 to 1.9). Furthermore, in the placebo arm, a reduction in subclinical inflammation beyond the SDC was not associated with statistically significant concurrent improvements in HAQ, morning stiffness duration or general well-being, in contrast to the observations made in the treatment arm.

## Discussion

The TREAT EARLIER trial showed that a 1-year course of methotrexate improved the severity of subclinical joint inflammation and related symptoms and physical impairments.^8^ We advanced on this by showing that, at the end of the 1-year treatment period, the group-level improvement in subclinical joint inflammation was predominantly driven by a subgroup (of 38%) of well-responding CSA patients. This indicates that when the aim is to reduce the inflammatory disease burden in patients with CSA and subclinical joint inflammation in a way that avoids overtreatment, this subgroup of patients should be treated preferentially. These responding patients can be recognised by increased levels of subclinical inflammation at baseline.

In this work, we specifically identified the presence of more extensive tenosynovitis (two or more tendon sheaths) with or without osteitis as a strong predictor of treatment response. Tenosynovitis has previously been found to be associated with the severity of disability and also fist strength measures in CSA, both when assessed at physical examination or with a dynamometer.^1 17^ Osteitis was also a predictor for treatment response here, while osteitis has not been observed to associate with symptoms and disability in previous studies in CSA.^21 22^ Interestingly, patients with CSA and a positive MRI showing mainly subclinical synovitis did not respond well to methotrexate. This aligns with recent evidence in which tenosynovitis has been recognised as a marker whose presence is useful in assessing the risk of RA.^23^ This study shows that multiple inflamed tendon sheaths are also a predictor of a good response in inflammatory burden. Patients with CSA and with synovitis only and without tenosynovitis may still have a high disease burden. Alternative therapeutic approaches may be considered in these patients.^17 18^

We define treatment response based on MRI-detected subclinical inflammation because it provides an objective and disease-specific assessment of treatment efficacy. Previous studies have shown that MRI-detected subclinical joint information is related to joint tenderness, morning stiffness, fist strength and functional disability in the CSA phase.^17^,^19^ We used the SDC in RAMRIS inflammation scores to discern small fluctuations that could have been caused by measurement variability, regression to the mean and noise from a ‘true’ reduction of subclinical inflammation. This approach accounts for interobserver differences in MRI scoring, thereby improving reliability and reproducibility. By setting the SDC as a threshold, we minimised the risk of overestimating treatment effects, ensuring robust and reproducible results.

Furthermore, in a sensitivity analysis, we defined treatment response not as a reduction beyond the SDC on either tenosynovitis, synovitis or osteitis, but as a reduction of total inflammation score beyond the SDC, setting an even stricter threshold for defining treatment response. Here, the same predictive variables were identified, demonstrating the robustness of the results. Moreover, patients with a reduction in subclinical inflammation above the SDC showed greater improvements in physical functioning, pain and morning stiffness than those without a response, further reinforcing its validity as a definition for treatment response.

It could be argued that including MRI variables as an independent variable and outcome could lead to circularity; increased baseline subclinical inflammation might naturally predict its own reduction. However, the MRI predictors in our analyses specifically captured tenosynovitis and osteitis, whereas the outcome reflected the overall reduction of subclinical inflammation, without direct incorporation of synovitis scores. Moreover, we showed that MRI-defined treatment response was accompanied by clear improvements in patient-reported outcome measures, such as HAQ-DI, beyond the minimally clinically important improvement, demonstrating its utility as a meaningful indicator of disease burden independent of circular reasoning.^24^ Additionally, when repeating the multivariable logistic regression analysis in the placebo arm, baseline tenosynovitis scores were not significantly associated with treatment response, nor did we find significant differences between HAQ changes after 1 year in those with treatment response and without. This further validates that the observed MRI improvement reflects a true biological treatment response, and arthralgia individuals responding better to methotrexate can be identified by their extended levels of tenosynovitis at baseline.

Interestingly, clinical variables at baseline did not show an independent significant association with treatment response. Functional disability and morning stiffness are known to be associated with subclinical inflammation, but, by proxy, may have been less potent in predicting treatment response than the imaging variables themselves. Some laboratory variables (such as ACPA, RF and CRP) were considered and were not associated with treatment response. However, it is possible that other markers of systemic inflammation would be found relevant for predicting treatment response, if a wide array of inflammatory markers had been measured. Performing such work is an important topic for future research.

The TREAT EARLIER trial had two main objectives: first, to assess whether RA development could be intercepted with a 12-month methotrexate treatment, and second, to determine whether methotrexate could reduce the disease burden. In the present study, we focused on the latter since patients have indicated that improvements in functional disability, pain and morning stiffness are as relevant outcomes as prevention of the RA diagnosis itself.^25^ Therefore, reduction of inflammatory burden can be a treatment aim. Since previous analyses of the TREAT EARLIER trial showed an effect of treatment compared with placebo in HAQ after 2 years in the total trial population (and in ACPA-positive and ACPA-negative patients with an increased risk),^8 25^ a subsequent question was which patients can best be treated, since an effect seen in the total population may actually be based on an effect in a subgroup of patients. Indeed, we here showed that a subgroup of patients had the best response during the year of treatment. A next question concerns the durability of the effect after the year of treatment. The two earlier reports of the TREAT EARLIER trial showed that improvements in HAQ during the first year of treatment were sustained during the second year.^8 12^ Whether improvements in these symptoms and physical impairments are sustained until the end of follow-up after 5 years remains ongoing work.

At-risk populations are intrinsically more heterogeneous than populations of patients with classified RA. In subanalyses, we continued with a recent observation that the trial population was heterogeneous in risk and pathophysiology (as expressed by ACPA status).^12^ Here, we reduced heterogeneity by excluding patients who were retrospectively at low risk for RA (as patients not at risk for a disease cannot benefit from preventive treatment). In these subanalyses, investigating only patients with a predicted increased risk, the percentage of responding patients increased as well compared with the total trial population: while MRI-based treatment response was achieved in 38% of the total trial population, the corresponding rates were 37% and 66% in the ACPA-positive and ACPA-negative increased risk groups, respectively. Thus, in both ACPA groups, patients with a good treatment response could be identified with inflammation of two or more tendon sheaths or a combination of tenosynovitis and osteitis.

Based on a combination of the above-mentioned findings, precision medicine in increased-risk patients can therefore consist of several steps. First, individuals with a low risk can be identified, as no treatment benefit is expected in these patients regardless of outcome. Thereafter, if treatment is considered, the therapeutic aim could be assessed in shared decision-making. When the aim is to reduce the inflammatory burden, patients presenting with tenosynovitis, with or without concurrent osteitis, may be preferred for treatment, to minimise the risk of overtreatment. This could either influence clinical practice or future trials in this area.

Our results show that it is valuable to perform an MRI prior to treatment initiation. As discussed above, MRI-detected subclinical joint inflammation is also an important predictor for RA development.^26^ Thereafter, the information provided by MRI can be useful to predict a reduction in subclinical joint inflammation. The combination of these findings implies that it is valuable to perform MRI in patients presenting with CSA, as it provides information on the risk of RA and the chance of benefiting from methotrexate in terms of inflammatory burden.

Conventional contrast-enhanced MRI has several disadvantages: it requires intravenous contrast, is costly and has long scan times. Although more readily available, ultrasound is known to have a lower sensitivity for detecting tenosynovitis than MRI and cannot detect osteitis.^2^ Therefore, MRI may not be easily replaced by ultrasound. Progress has been made in the field of imaging, and a new simplified fluid-sensitive MRI protocol has been developed using Dixon sequences. This method does not require intravenously injected gadolinium contrast, has shown high correlations in detecting joint inflammation with conventional contrast-enhanced MRI, is available at routine 3T MRI scanners and has a scan time of 5 min. Consequently, the costs will be considerably lower than those of conventional MRI.^27^

In conclusion, treatment response at the level of subclinical inflammation above the SDC was observed in 38% of patients treated in the TREAT EARLIER trial. Patients who responded best to methotrexate can be recognised by increased levels of subclinical inflammation at diagnosis, specifically with several sites of tenosynovitis and osteitis. These data may contribute to personalised medicine for individuals with arthralgia at risk for RA.

## Supplementary material

10.1136/rmdopen-2025-006102 online supplemental file 1

## Data Availability

Data are available on reasonable request.
