# Supplementary material for: Which arthralgia patients benefit most in reduction of subclinical joint inflammation by methotrexate treatment: results from the TREAT EARLIER trial
Source: RMD Open. 2026 Jan 20;12(1):e006102. doi: 10.1136/rmdopen-2025-006102 (PMC12820865; doi:10.1136/rmdopen-2025-006102)
Supplement: online supplemental file 1 [file rmdopen-12-1-s001.docx]

Supplementary file

Table of Contents

[**Supplementary file 1**. Participants 2](#_Toc213675913)

[**Supplementary file 2**. Randomisation and masking 4](#_Toc213675914)

[**Supplementary file 3**. MRI imaging and scoring protocols 5](#_Toc213675915)

[**Supplementary file 4.** Interpretation of subclinical inflammation change scores 8](#_Toc213675916)

[**Supplementary file 5**. Venn diagram 9](#_Toc213675917)

[**Supplementary file 6.** Sensitivity analysis with an alternative definition of treatment response based on the smallest detectable change of the sum of subclinical inflammation scores using RAMRIS. 10](#_Toc213675918)

[**References of supplementary file** 12](#_Toc213675919)

## **Supplementary file 1**. Participants

**Participants**

Adults aged 18 years or older with arthralgia at risk of developing rheumatoid arthritis were eligible for enrolment across 13 rheumatology outpatient clinics in the southwest region of the Netherlands. We used a two-level definition to identify patients predisposed to develop rheumatoid arthritis. First, patients needed to have recent-onset (within the past year) arthralgia that was suspected of progressing to rheumatoid arthritis according to the treating rheumatologist (i.e., clinically suspect arthralgia). Second, an MRI scan of their hands or forefeet had to show subclinical joint inflammation.

Clinically suspect arthralgia, a complex of clinical symptoms and signs, was identified by rheumatologists using pattern recognition, as no single symptom is sufficiently specific for imminent rheumatoid arthritis. By definition, clinically suspect arthralgia was not present if patients presented with clinical arthritis or if another explanation for the symptoms was more likely to be the cause (e.g. , osteoarthritis, fibromyalgia, or suspicion of developing psoriatic arthritis or gout). Establishing clinically suspect arthralgia does not require abnormal results on laboratory investigations (e.g. , acute phase reactant or autoantibodies). Compliance with the European Alliance of Associations for Rheumatology definition of arthralgia at risk of rheumatoid arthritis was not mandatory, as it was not yet developed at the start of the trial.

To screen for the second inclusion criterion, we invited all patients in the region identified as having clinically suspect arthralgia for a contrast-enhanced 1·5T extremity MRI of the metacarpophalangeal, wrist, and metatarsophalangeal joints. MRIs were assessed by two readers independently for subclinical inflammation (i.e., synovitis, tenosynovitis, or osteitis), using the rheumatoid arthritis MRI scoring method (appendix pp 2–4). Subclinical inflammation was defined as present if at least one joint showed one or more inflammatory features scored by both readers and that were present in fewer than 5% of age-matched symptom-free volunteers at the same location. If only one reader identified the presence of such inflammation, the MRI was considered negative (appendix p 4). Readers were masked to any clinical data and showed excellent intrareader and inter-reader reliability (intraclass correlation coefficients >0·90). To speed up screening, immediately after scanning, MRIs were scored and patients were informed on the presence or absence of subclinical inflammation

We excluded patients with (history of) clinical arthritis, previous or current treatment with DMARDs or glucocorticoids, contraindications for MRI, pregnancy or breastfeeding, bone marrow hypoplasia, elevated hepatic enzyme concentrations (>3 times the upper normal limit), serum creatinine concentration of greater than 150 μmol/L or estimated clearance of less than 60%, serious infections in the past 3 months, or chronic infectious diseases.

Written informed consent for the MRI was obtained from all patients screened. Subsequently, patients who met the inclusion criteria and were willing to participate provided written informed consent for the complete trial.[1]

## **Supplementary file 2**. Randomisation and masking

**Randomisation and masking**

The hospital trial pharmacist randomly assigned participants (1:1) to active treatment or placebo (using computer-generated block randomisation [block size of ten] without stratification) and issued all study medication, but had no further involvement in the trial. The appearance, packaging, and distribution of the intramuscular glucocorticoid injection and methotrexate tablets were identical to the corresponding placebo products. Neither the participants nor the treating rheumatologist, study team, or staff involved had any knowledge on which treatment participants would receive, ensuring allocation concealment. All participants and staff involved (including those administering study medication, assessing endpoints, and analysing the data) were masked to group allocation until after database lock. MRI results during follow-up were not communicated to participants nor to staff involved with data collection or treatment decisions.[1]

## **Supplementary file 3**. MRI imaging and scoring protocols

***Imaging protocol***

MRI was performed on an MSK-extreme 1.5T extremity MRI system (GE, Wisconsin, USA) using a 145mm coil for the foot and a 100mm coil for the hand. The patient was positioned in a chair beside the scanner, with the hand or foot fixed in the coil with cushions.

In the hand (metacarpophalangeal (MCP) joints 2-5 and wrist) the following sequence was acquired before contrast administration: T1 weighted fast spin-echo (FSE) sequence in the coronal plane (repetition time (TR) 575 ms, echo time (TE) 11.2 ms, acquisition matrix 388×288, echo train length (ETL) 2). After intravenous injection of gadolinium contrast (gadoteric acid, Guerbet, Paris, France, standard dose of 0.1 mmol/kg) the following sequences were obtained: T1-weighted FSE sequence with frequency selective fat saturation (fatsat) in the coronal plane (TR/TE 700/9.7ms, acquisition matrix 364×224, ETL 2), T1 weighted FSE fatsat sequence in the axial plane (wrist: TR/TE 540/7.7 ms; acquisition matrix 320x192; ETL 2 and MCP-joints: TR/TE 570/7.7 ms; acquisition matrix 320x192; ETL 2). For the forefoot, these were: T1-weighted FSE fatsat sequence in the axial plane (TR/TE 700/9.5ms; acquisition matrix 364x224, ETL 2) and: T1-weighted FSE fatsat sequence in the coronal plane (perpendicular to the axis of the metatarsals) (TR/TE 540/7.5ms; acquisition matrix 320x192, ETL 2).

Field-of-view was 100mm for the hand and 140mm for the foot. Coronal sequences of the hand had 18 slices with a slice thickness of 2mm and a slice gap of 0.2mm. Coronal sequences of the foot had 20 slices with a slice thickness of 3mm and a slice gap of 0.3mm. All axial sequences had a slice thickness of 3mm and a slice gap of 0.3mm with 20 slices for the wrist, 16 for the metacarpophalangeal-joints and 14 for the foot.

We used the contrast enhanced T1-weighted fat suppressed sequence to assess osteitis in the wrist-, MCP-, and MTP-joints of all patients. According to the RAMRIS-method, T2-weighted fat suppressed sequences, or when this sequence is not available a short tau inversion recovery (STIR) sequence, should be used to assess osteitis. However, three previous studies have demonstrated that a contrast enhanced T1-weigthed fat suppressed sequence has a strong correlation with T2-weighted fat suppressed sequences.[1-3] Furthermore, the arthritis subcommittee of the European Society of Musculoskeletal Radiology (ESSR) also recommends the use of contrast enhanced T1-weighted fat suppressed sequences for depicting osteitis.[4] The T2-weighted image shows increased water signal and a contrast-enhanced T1 weighted sequence shows increased water content and the increased perfusion and interstitial leakage. A strong correlation has been shown in arthritis patients and in patients without inflammatory diseases such as bone bruises, intraosseous ganglions, bone infarcts and even nonspecific cases.[2, 3] Based on these results osteitis was assessed on contrast enhanced T1- 2 weighted fat suppressed sequences as it has a higher signal to noise ratio and allowed a shorter scan time for patients. In addition, because T2-weighted fat suppressed sequences could be omitted, coronal sequences of the foot could be added. In total this resulted in a shorter total scan time and more information. This scanning protocol has also been used over the past decade for the preliminary observational work in CSA and healthy controls preceding the design of this trial.

*MRI readers*

The MRIs were scored directly after the MRI was made. Two readers scored the images independently, and blinded for all clinical data. These readers were part of a group of eleven readers who took turn to be readily available for scoring MRIs of possibly eligible participants of the TREAT EARLIER-trial. Serial MRI’s were scored by two different readers (A and B). All readers underwent an extensive training period before scoring the trial screening MRIs. Interreader ICC of reader 1-11, A and B after this training period, and their intra-reader correlation coefficients after 6 months (ICCs) were all excellent (>0.90).

*MRI scoring*

All bones, joints and tendon sheaths were scored semi-quantitatively according to the validated RA MRI scoring system (RAMRIS). Osteitis was scored on a scale 0-3 based on the affected volume of the bone (no osteitis, >0-33%, >33-66%, >66%) and synovitis was scored on a range 0-3 based on the volume of enhancing tissue in the synovial compartment (none, mild, moderate, severe)[5]. Similar to methods described by Haavardsholm et al. the tenosynovitis-score was based on the thickness of peritendinous effusion or synovial proliferation with contrast enhancement (normal, 5mm (range 0-3))[6]. Osteitis was scored in the proximal and distal MCP 2-5 (8 bones) and, proximal and distal MTP 1-5 (10 bones), and in 13 bones in the wrist. In the wrist the carpometacarpal-1 joint (base of metacarpal 1 and the trapezium) were excluded, since these are considered osteoarthritis locations.[7] The proximal and distal bones of MCP- and MTPs were summed per joint. Therefore, 22 locations will be assessed for osteitis: 4 for the MCPs, 5 for the MTPs (both with a range of 0-6 per joint) and 13 for the wrist (with a range of 0-3 per bone). Synovitis was assessed in 12 joints: MCP2-5, MTP1-5, and in three regions of the wrist. Tenosynovitis was scored in 18 tendon sheaths: 10 tendon sheaths in the wrist and 8 for the MCPs (4 flexor and 4 extensor tendon sheaths). Thus, the total tenosynovitis score was a sum of 18 tendon sheaths.

*Definition of presence of subclinical inflammation on MRI*

The definition of presence of subclinical inflammation on MRI was only used to determine which screened participants were eligible for inclusion in the TREAT EARLIER-trial. This definition was not used for the analyses of the course of MRI-detected joint inflammation. Per location, scores of osteitis, synovitis and tenosynovitis were compared to the scores of symptom-free controls at the same location in the matching age-group (60 years old)[7]. For this, volunteers were recruited between November 2013 – December 2014 from the general population in Leiden, The Netherlands via advertisements on websites and local newspapers. Via telephone, people were screened for the inclusion criteria age ≥ 18, no history of inflammatory rheumatic disease and absence of joint symptoms during the last month. Subsequently, the presence of clinical arthritis was excluded via physical examination of the hands and feet at the outpatient clinic. In total 193 symptom-free controls underwent contrast-enhanced MRIs of the hands and forefeet which were scored for the presence of synovitis, tenosynovitis and osteitis, described previously.[1] For participants eligible for inclusion in the TREAT EARLIER trial, presence of subclinical inflammation on MRI of was considered positive per location if each of the two readers independently indicated presence of the inflammatory lesion, and in addition this lesion was present in <5% of the symptom-free controls in the same age-category. [7,8] An MRI was considered positive if a patient had at least one positive lesion as described. If only one reader identified the presence of an inflammatory lesion (that is the same location present in <5% of symptom-free controls in the same age-category) the MRI was considered negative for subclinical inflammation [7,8].

Serial MRI’s that were made on baseline and during follow-up were scored by reader A and B with known time-order and blind for clinical characteristics or knowledge of study endpoints or treatment allocation [9]. Total osteitis, synovitis, and tenosynovitis scores (mean of the two readers) were summed into the MRI-detected inflammation.

## **Supplementary file 4.** Interpretation of subclinical inflammation change scores

**Figure S4.** Interpretation of subclinical inflammation change scores


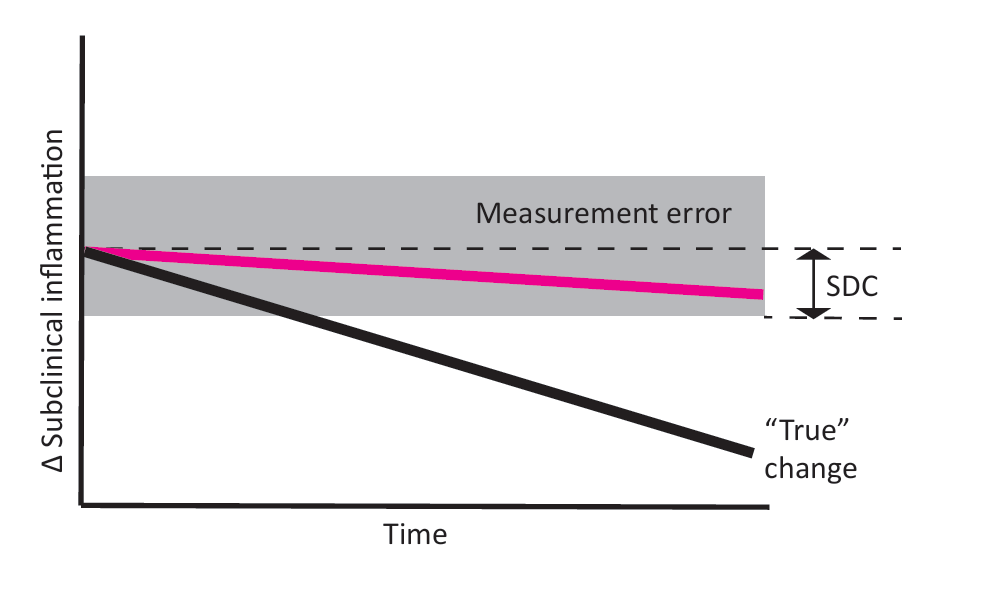


*Legend:* When using continuous measurements of subclinical inflammation over time, change scores have to be interpreted carefully. Smaller changes can be attributed to variations in measurement and noise, whereas larger changes are meaningful to the patient’s condition. In order to differentiate between these changes, the smallest detectable change can be calculated (SDC) for both positive and negative changes. A change greater than the SDC can be considered a “true change”.

## **Supplementary file 5**. Venn diagram

**Figure S5.** Venn diagram displaying on which MRI features patients with treatment response reached the cut-off of reduction beyond the smallest detectable difference


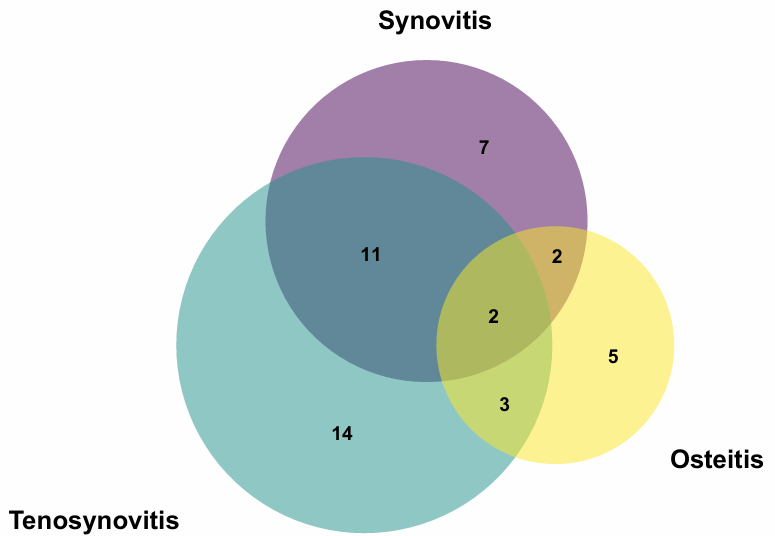


## **Supplementary file 6.** Sensitivity analysis with an alternative definition of treatment response based on the smallest detectable change of the sum of subclinical inflammation scores using RAMRIS.

**Table S6.1** Multivariable logistic regression analysis of baseline characteristics for association with MRI-defined treatment response using the outcome SDC total (sensitivity analysis)

| MRI characteristics | | Odds ratio (95 % CI) |
| --- | --- | --- |
| Tenosynovitis | 0 | Reference |
| (per tendon sheath) | 1 | 7.2 (1.3-41) |
|  | 2 | 12 (2-76) |
|  | > 3 | 157 (22-1100) |
| Osteitis | 0 | Reference |
| (per bone) | 1 | 1.8 (0.4-7) |
|  | > 2 | 23 (3-168) |

**Table S6.2** Combinations of tenosynovitis and osteitis show a strong association with SDC total treatment response outcome

| Combinations of MRI characteristics | Odds ratio (95 % CI) |
| --- | --- |
| Osteitis > 1 & Tenosynovitis > 1 | 6.8 (2.3-20)* |
| Osteitis > 2 & Tenosynovitis > 1 | 13.8 (1.5-123)* |
| Osteitis > 1 & Tenosynovitis > 2 | 16.5 (3.3-80)* |
| Osteitis > 2 & Tenosynovitis > 2 | Infinite (0-Infinite)* |

*= Statistically significant beyond the 5 per cent level

**Table S6.3** Positive and negative predictive values of the number of tenosynovitis and osteitis locations, using the outcome SDC total

| Characteristics | Positive predictive value  Sensitivity analysis (SDC total) | Negative predictive value  Sensitivity analysis (SDC total) |
| --- | --- | --- |
| Tenosynovitis of one or more locations | 46% | 92% |
| Tenosynovitis of two or more locations | 62% | 87% |
| Tenosynovitis of three or more locations | 86% | 84% |
| Osteitis of two or more locations | 67% | 75% |
| Combination of osteitis and tenosynovitis† | 79% | 77% |
|  |  |  |

†Combination of osteitis and tenosynovitis, where at least one involves two or more sites

Of note, 1 of the patients that was classified as treatment response in this analysis (using the SDC total outcome) did progress to RA. This was an ACPA-positive patient who developed clinically apparent arthritis in the MCP2 joint, while the sum of tenosynovitis and osteitis scores decreased beyond the smallest detectable change of the sum of subclinical inflammation score (SDC total). Reclassifying this patient to the group without treatment response yielded similar results (data not shown).

## **References of supplementary file**

1. Krijbolder, D.I., Verstappen, M., Van Dijk B.T, et al., Intervention with methotrexate in patients with arthralgia at risk of rheumatoid arthritis to reduce the development of persistent arthritis and its disease burden (TREAT EARLIER): a randomised, double-blind, placebo-controlled, proof-of-concept trial. Lancet, 2022. 400(10348): p. 283-294.

2. Stomp W, Krabben A, van der Heijde D, et al. Aiming for a shorter rheumatoid arthritis MRI protocol: can contrast-enhanced MRI replace T2 for the detection of bone marrow oedema? Eur Radiol. 2014;24(10):2614-22.

3. Schmid MR, Hodler J, Vienne P, Binkert CA, Zanetti M. Bone marrow abnormalities of foot and ankle: STIR versus T1-weighted contrast-enhanced fat-suppressed spin-echo MR imaging. Radiology. 2002;224(2):463-9.

4. Mayerhoefer ME, Breitenseher MJ, Kramer J, Aigner N, Norden C, Hofmann S. STIR vs. T1 weighted fat-suppressed gadolinium-enhanced MRI of bone marrow edema of the knee: computer-assisted quantitative comparison and influence of injected contrast media volume and acquisition parameters. J Magn Reson Imaging. 2005;22(6):788-93.

5. Sudoł-Szopińska I, Jurik AG, Eshed I, et al. Recommendations of the ESSR Arthritis Subcommittee for the Use of Magnetic Resonance Imaging in Musculoskeletal Rheumatic Diseases. Semin Musculoskelet Radiol. 2015;19(4):396-411.

6. Østergaard M, Peterfy C, Conaghan P, et al. OMERACT Rheumatoid Arthritis Magnetic Resonance Imaging Studies. Core set of MRI acquisitions, joint pathology definitions, and the OMERACT RA-MRI scoring system. J Rheumatol. 2003;30(6):1385-6.

7. Haavardsholm EA, Østergaard M, Ejbjerg BJ, Kvan NP, Kvien TK. Introduction of a novel magnetic resonance imaging tenosynovitis score for rheumatoid arthritis: reliability in a multireader longitudinal study. Ann Rheum Dis. 2007;66(9):1216-20.

8. Mangnus L, van Steenbergen HW, Reijnierse M, van der Helm-van Mil AH. Magnetic Resonance Imaging-Detected Features of Inflammation and Erosions in Symptom-Free Persons From the General Population. Arthritis Rheumatol. 2016;68(11):2593-2602.

9. Boer AC, Burgers LE, Mangnus L, et al. Using a reference when defining an abnormal MRI reduces false-positive MRI results-a longitudinal study in two cohorts at risk for rheumatoid arthritis. Rheumatology (Oxford). 2017;56(10):1700-1706.
